# The reduction of intoxication and disorder in premises licensed to serve alcohol: An exploratory randomised controlled trial

**DOI:** 10.1186/1471-2458-10-607

**Published:** 2010-10-14

**Authors:** Simon C Moore, Iain R Brennan, Simon Murphy, Ellie Byrne, Susan N Moore, Jonathan P Shepherd, Laurence Moore

**Affiliations:** 1 Violence & Society Research Group, Cardiff School of Dentistry, Cardiff University, Cardiff, CF14 4XY, UK; 2 Cardiff Institute for Society and Health, School of Social Sciences, Cardiff University, 1-3 Museum Place, Cardiff, CF10 3BD, UK

## Abstract

**Background:**

Licensed premises offer a valuable point of intervention to reduce alcohol-related harm.

**Objective:**

To describe the research design for an exploratory trial examining the feasibility and acceptability of a premises-level intervention designed to reduce severe intoxication and related disorder. The study also aims to assess the feasibility of a potential future large scale effectiveness trial and provide information on key trial design parameters including inclusion criteria, premises recruitment methods, strategies to implement the intervention and trial design, outcome measures, data collection methods and intra-cluster correlations.

**Design:**

A randomised controlled trial in licensed premises that had experienced at least one assault in the year preceding the intervention, documented in police or hospital Emergency Department (ED) records. Premises were recruited from four study areas by piloting four recruitment strategies of varying intensity. Thirty two licensed premises were grouped into matched pairs to reduce potential bias and randomly allocated to the control or intervention condition. The study included a nested process evaluation to provide information on intervention acceptability and implementation. Outcome measures included police-recorded violent incidents, assault-related attendances at each premises' local ED and patron Breath Alcohol Concentration assessed on exiting and entering study premises.

**Results:**

The most successful recruitment method involved local police licensing officers and yielded a 100% success rate. Police-records of violence provided the most appropriate source of data about disorder at the premises level.

**Conclusion:**

The methodology of an exploratory trial is presented and despite challenges presented by the study environment it is argued an exploratory trial is warranted. Initial investigations in recruitment methods suggest that study premises should be recruited with the assistance of police officers. Police data were of sufficient quality to identify disorder and street surveys are a feasible method for measuring intoxication at the individual level.

**Trial registration:**

UKCRN 7090; ISRCTN: 80875696

**Funding:**

Medical Research Council (G0701758) to Simon Moore, Simon Murphy, Laurence Moore and Jonathan Shepherd

## Background

Exposure to the night time economy (NTE) environment is associated with an increased likelihood of violent victimisation [1]. Approximately 47% of assaults against adults in England and Wales are committed by offenders believed to be under the influence of alcohol, with a large proportion of incidents taking place at weekends in urban centres [2]. Managing public alcohol use and preventing alcohol-related harm are priorities in numerous jurisdictions across the world and have motivated both policy-level (e.g. the UK 2003 Licensing Act) and individual-level interventions [3]. Accordingly, NTE licensed premises are increasingly the subject of violence prevention initiatives [4]. Legislation on alcohol-related harm and disorder typically focuses on situational preventive measures, such as opening hours regulation, staff training, enforcing the refusal of service to intoxicated patrons, and the replacement of drinking glasses and bottles with plastic alternatives. Such approaches have gained considerable traction across practitioner groups including police, local authority licensing staff and health professionals. In light of the considerable costs due to alcohol-related disorder it is essential that preventive methods are supported by robust evidence. However, few formal evaluations of premises-level interventions have been conducted, and none in the UK [4,5]. Furthermore, it is unclear whether intervention delivery should be targeted at those premises generating the greatest levels of harm or all premises in a NTE. If interventions are to be targeted at problematic premises, it is not clear how these premises should be identified and how they should be recruited into evaluation studies.

Premises-specific risk factors for disorder can be easily identified and therefore managed. Recent legislation that has made premises managers accountable for managing risk in their premises (UK 2003 Licensing Act) has meant that harm and disorder programmes have typically focused on the premises environment rather than the more general NTE environment. The reasoning for this is that the identification and manipulation of situational risk factors can influence the likelihood of an undesirable outcome - a risk factor prevention paradigm. Although a number of studies have identified those characteristics of licensed premises that are associated with harm and disorder [6-8], optimal methods for targeting, recruiting and intervening have not been adequately described in the UK.

Previous evaluations have considered interventions such as responsible beverage server (RBS) training, licensee accords and staff violence reduction training. RBS training, the most commonly evaluated intervention type, typically deploys "off-the-shelf" training packages that do not involve any consideration of premises' underlying risk factors. These unfocused interventions are likely to be less effective than interventions that are responsive to the risks and needs of individual premises. Of the available RCT evaluations that have been conducted in this area, only Graham et al. [9] implemented an intervention that was responsive to the idiosyncratic needs of premises, while Toomey et al. [10] evaluated a not dissimilar risk-led intervention using quasi-experimental methods. Both of these studies concluded that premises-level interventions that are designed to offset risk factors in each premises are feasible.

A range of outcome measures have been used in premises-level evaluations, including police records, rates of hospital treatment following violent injury and customer breath alcohol concentration as well as subjective ratings such as customer self-report and observations of disorder and intoxication by research staff. While convenient, subjective ratings can be susceptible to response and reporting biases. Police records follow a standardised recording protocol, especially after the implementation of a national crime recording standard in England and Wales, that makes them more reliable and appropriate for studying changes in crime over time. As these data also contain information on the location of incidents they can be linked to individual premises. However, police data are susceptible to systematic bias as usually only those incidents that are reported to the police or occur when the police are present are recorded. Emergency Department (ED) data are not susceptible to such biases as serious injury will require hospital treatment irrespective of where and when the assault took place. However, this does mean that ED data are biased towards more serious assaults. ED data are usually collected by reception staff who record details of violence location, time, day and weapon. If the patient declares that their injury is assault-related then this prompts a series of questions about the nature of the incident [11]. In respect of premises proclivity to sell alcohol inappropriately, customer intoxication can be assessed using subjective measures but are inferior to objectively recorded Breath Alcohol Concentration (BrAC) collected using alcometers [12-14].

Of the existing evaluations of premises-level interventions, only three have used a RCT design to measure intervention effectiveness in terms of objectively measured outcomes of intoxication [15,16] or disorder [9]. Furthermore, when matching procedures were used for these studies, they were limited to "bar type" or premises size and failed to consider the past history of alcohol-related harm and opening hours - characteristics that may also predict levels of violence and intoxication. Moreover, past trials have failed to account for the displacement or diffusion effects of premises-level interventions which may undermine evaluations of intervention effectiveness.

This paper reports upon the design of an exploratory trial of a targeted, risk factor-focused intervention, the Licensed Premises Harm Reduction Initiative (LPHRI), which identifies high risk premises using a risk factor prevention approach designed to reduce alcohol intoxication and disorder in licensed premises. Details of the intervention are now described.

## The Licensed Premises Harm Reduction Initiative intervention

• High risk premises are identified through the analysis of routine data (police and ED recorded assaults)

• Premises risk factors for intoxication and disorder are assessed using an audit consisting of two premises walkthroughs (day and night) and one face to face interview with the premises manager. Major categories of risks cover the external environment immediate to the premises, the staff, customer behaviour, the internal physical environment, operational procedures, and security measures.

• Results from the audit inform a bespoke premises action plan, delivered to premises managers in the experimental condition only, that identifies risk factors and suggests solutions. Premises managers will be telephoned one week later to ensure the action plan had been received.

• A second audit, identical to the first, is delivered three months later to assess distance travelled and to provide feedback.

Premises-level interventions have not been trialled in the UK and therefore effect sizes are unknown, prompting the need for an exploratory trial. This study aimed to identify optimum methods for identifying and recruiting premises, the development of a sustainable and rigorous evaluation methodology, the description of matching and randomisation procedures, the identification of appropriate outcome measures, and an understanding of intervention implementation. Particular challenges present themselves in identifying and recruiting high risk premises to such studies; these are outlined and their implications for any definitive RCT of the intervention are discussed.

The study sought to identify methods for intervention targeting, facilitating trial recruitment, developing and maintaining rigorous research designs and identifying and collecting appropriate outcome measures. The qualitative nested process evaluation examined views on intervention theory and acceptability, implementation processes and fidelity, assessed potential contamination between trial arms and identified the structures required for any definitive trial.

## Methods/Design

### Study design

**Figure 1:**
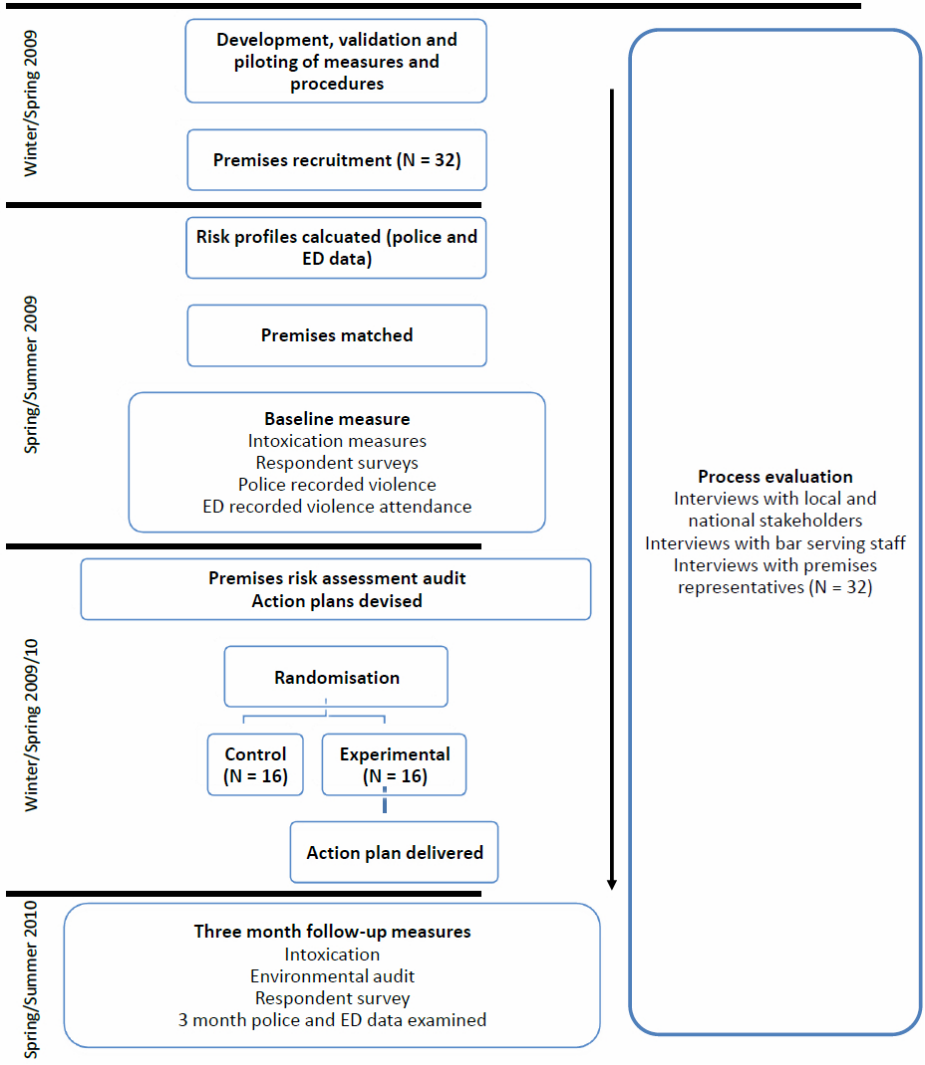
**Study Design**.

Figure 1 provides a summary of the study, a mixed methods exploratory RCT with nested process evaluation. All aspects of this study were evaluated and approved by the Cardiff University Medical and Dental School Research Ethics Committee (Ref: MDSREC 08/11). Methods were informed by a previous study examining alcohol misuse and violence in the NTE funded by the Alcohol Education Research Council (CA04/01) [13,14].

### Recruitment

As the characteristics of NTEs and the violence prevention resources available can vary considerably across jurisdictions, the study team implemented the intervention across five towns (reduced to four areas for analytical purposes as two towns, D and E in Table 1, are in close proximity and are typically referred to as an homogenous region). The four areas covered by the study were selected as they featured NTE types representative of those in England and Wales, including cities and small to medium-sized towns.

**Table 1:** Recruited premises by recruitment intensity

Recruitment Strategy	Town A	Town B	Town F	Town D	Town E	Town C
Resident population	325,000	227,000	50,000	47,000	35,600	37,000
**Number of eligible licensed premises**	**89**	**81**	**30**	**11**	**9**	**10**
**Invitation letters sent**	89	81	30	26		
**Expression of interest**	4	2	1	1		
**Recruited**	2	1				

**Premises managers forum**						
**Number of eligible licensed premises**	30	25	10			
**Expression of interest**	2	3				
**Recruited**	1	3				

**Premises visits with police**						
**Number of premises targeted**						6
**Recruited**						6

**Premises visits, no police**						
**Number of premises targeted**	2	7		6	2	2
**Recruited**	2	7		6	2	2

A variety of recruitment strategies that varied in intensity were piloted in phases across the intervention areas. Local police in each area were aware of and supportive of the project. However, they were not involved either in the design or evaluation of the intervention. While it was desirable that police involvement be kept to a minimum in order to facilitate transparency, it was concluded that local police licensing officers represent the most efficient route for accessing and recruiting licensed premises managers. Therefore, licensing officers were involved in the recruitment methodology. Premises were first invited to participate by letter, following which researchers made appeals at premises manager meetings. Once these first two options were exhausted, visits to premises were made by Project staff. All visits involved police officers, either directly through accompanying researchers or indirectly through officers introducing the study to premises staff beforehand. This order was maintained where possible, however due to the timing of some local meetings, the availability of premises staff and Project time constraints, it was not possible to follow this order of events strictly in each area. Irrespective of a premises pathway into the study all premises were visited by a member of the research team before the study commenced in order to describe the project in detail, to allow representatives to ask questions, and to gain verbal consent. Each premises representative was given a detailed description of the trial and what would be required of them.

### Inclusion/exclusion criteria

Premises were eligible for inclusion if (1) a police-recorded violent offence took place in or immediately outside the premises in the twelve months before recruitment, (2) or an attendance at an ED for violent injury was associated with the premises or (3) the premises was identified as being at high risk of disorder by the local police licensing officer. It is likely that police records represent a conservative estimate of the number of serious violent incidents in and around the target premises as it has been demonstrated that the majority of serious violent incidents are not reported to the police [17], but police data are likely to reflect information on incidents where no ED attendance is required, suggesting that a composite dataset is appropriate.

### Matching

The unit of allocation was the individual licensed premises, stratified by risk of harm and area. A risk index was calculated for each licensed premises using the formula Risk = P/(T*C) where P is the number of police recorded incidents in the twelve months preceding project start, T is the number of hours open after 11 pm on Friday and Saturday night and C is the maximum premises capacity. Matching was incorporated into the study to reduce possible imbalances between treatment arms expected with a small sample size, but was not accounted for in subsequent analyses due to the loss of degrees of freedom [18].

### Randomisation

The exploratory trial was a two-armed parallel cluster randomised trial in which premises was the unit of randomisation. Only once the audits and action plans for all thirty-two premises had been completed did randomisation take place. Each premises in a pair was assigned the number one or two in the order they were recruited by Researcher 1. Independently a random number was generated and relayed by telephone to Researcher 1. If an odd number was generated, the premises identity marked one was placed in an envelope and sealed. If an even number was generated, the premises identity marked two was placed in an envelope and sealed. Unselected premises names were placed out of sight. Researcher 1 left the randomisation room and was replaced by Researcher 2. A random number was independently generated and relayed by telephone to Researcher 2. If an odd number was generated, the premises identity in the envelope was allocated to the control group. If an even number was generated, the premises identity marked was allocated to the intervention group. Each premises had a 50% chance of being in either control or intervention group.

### Blinding

The research team members who were responsible for the delivery and evaluation of the intervention were blind to the intervention condition of each premises, as were all data collection staff. Bespoke intervention documents were prepared for all premises by a member of the intervention team although these documents were only delivered to intervention premises. These documents were prepared for delivery by a member of the university administrative staff who played no other part in the study. Since part of the process evaluation required asking different questions of intervention and control premises representatives, the research team members who conducted the process evaluation were necessarily unblinded.

### Measures

The outcome measures used in the study were selected for their appropriateness and objectivity in identifying and accurately associating violence, severe intoxication and environmental disorder with study premises. Identical outcome measures were used at baseline and at the three month follow-up to measure violence, severe intoxication and environmental disorder.

#### Police records

Police records of violence against the person inside or entering or leaving the premises were used as a measure of disorder. An incident was also deemed to be associated with a premises if a participant involved in the incident had been in the premises or attempted to enter the premises immediately prior to the incident.

#### Emergency Department data

Any attendance for treatment of violent injury that was recorded as such in ED records and was associated with a study premises was counted as a violent incident.

#### Environmental observations

Surveyors recorded levels of environmental disorder and contextual risk factors, as well as keeping a continuous record of the characteristics of clients entering and exiting study premises. These records also included details of specific disorder-related incidents, such as ejections from licensed premises, fights and arrests.

#### Patron survey

The survey questions covered respondent gender, age, marital status, employment status, smoking habits, drinking locations and number of people in their group that evening and their intended destination (named premises, home etc.). In addition, the survey incorporated the established Fast Alcohol Screening Test (FAST) [19] - a series of four questions often used to select individuals for brief interventions for alcohol misuse, and three items on respondent's experiences of violent victimisation and violent offending in the preceding twelve months.

#### Breath alcohol concentration

While the police record information on the circumstances of disorder and violence, objective measures of intoxication are not routine and were therefore collected by surveyors. Respondents were breathalysed using a Lion Laboratories SD-400 alcometer and had their BrAC recorded. Surveyors also responded to questions eliciting their subjective measure of drunkenness for respondents who did and did not provide a BrAC. These measures were used to assess potential sampling biases. Alcometers were recalibrated at least one every three months using the manufacturer's equipment and methods.

### Data collection

#### Police data

A data processing agreement between Cardiff University and South Wales Police facilitated data sharing. Data were encrypted and anonymised before transmission to Project staff who interrogated the data for instances of disorder in or around study premises. Incidents were then collated for analysis.

#### ED data

Information collected about patients who have attended an ED as a result of violent injury represents a valuable source of data on violence against the person [20]. Some EDs routinely record detailed information about the patient and the circumstances of the violent incident, including the location of the assault, the number of assailants and injury severity. However, this practice is not universal with some EDs simply recording victim information such as age and time of attendance. At baseline, detailed information about the location of assaults resulting in ED attendance was only available for Town A (see Table 1) across the duration of the baseline period. Data were available for Town B but only for a three-month period. No ED data were available for the remaining areas. A data sharing agreement between Cardiff University and hospitals local to study premises facilitated data sharing. Data for the available time periods were requested from the hospital information services. A Senior Information Analyst retrieved, anonymised and formated these data before transmission to Project staff who interrogated these data for instances of disorder in or around study premises. Incidents were then collated for analysis.

#### Street observations, surveys and breath alcohol concentration data

A surveyor standing four to five metres from the main entrance to a premises carried out environmental observations for the duration of the data collection episode. This involved the continuous observation of pedestrians moving to and from the study premises, a spot survey of environmental conditions every 30 minutes and recording and describing any disorderly or violent incident in the immediate area, such as ejections, fights and arrests. A second pair of surveyors recruited every seventh individual walking past a designated point near to the study premises and asked them to take part in a survey. If the person assented then the surveyor proceeded to ask the survey questions. All survey responses were completed by the surveyors on behalf of the respondents. On completion, respondents were asked to provide a BrAC reading using the alcometer. This was recorded. Once the respondent had left the vicinity, the surveyor rated them on the four descriptors used to identify drunkenness: gait, eyes and speech [14] and overall drunkenness along a 10-point Likert scale. All potential respondents who did not agree to participate were scored on the same subjective descriptors so that potential sampling biases could be assessed.

#### Data accuracy

Data from the baseline street surveys were entered by one data entry clerk. In order to assess the accuracy of data entry, a randomly selected sample of 5% were double checked by Project staff. An agreement level of 90% or better was determined as acceptable.

### Statistical analyses

In the available ED and police data concerning violence, it is not possible to determine whether multiple incidents on the same day are related or not: it is feasible that a single episode involving violence can lead to multiple arrests and/or injuries requiring hospital treatment. Furthermore, as the primary interest is premises-level risks it is reasonable to assume that premises failings persist across a session and therefore that multiple incidents in one session can be assumed as partly reflecting those risks. For violence, therefore, we assumed that one or more violent incidents indicated that for that session the premises was in a state of failure and was thus coded as a binary event. Furthermore, it is likely that any intervention effect will wane over time, particularly as premises are usually subject to high staff turnover rates. Moreover, for any definitive trial it is feasible that numerous external factors might influence premises-level failure rates, such as sporting events and temporary closure. While Poisson models can accommodate aggregate count data and would normally be suitable, in order to account for potential intervention wane, time varying covariates, censoring, multiple events and discontinuous risk intervals, the preferred approach was to develop an Andersen-Gill model [21], a derivation of the Cox proportional hazards model [22,23]. The individual level BrAC data are clustered in premises and location and is available pre- and post-intervention. This suggests that a multilevel mixed-effects linear regression [24] is appropriate from which design effects and intra-cluster correlation coefficients for BrAC and the other survey measures can be calculated.

### Sample size

Sixteen pairs of premises provide 80% power to detect a 0.8 standard deviation difference in premises-level rates of intoxication and violence using a two-tailed alpha of 0.05. The study was an exploratory trial and a significant effect was not anticipated.

### Process evaluation

A detailed process evaluation is being undertaken within the RCT evaluation. The project employs a framework described by Steckler and Linnan [25] to explore the implementation, fidelity and acceptability of the intervention. The aims of this process evaluation are twofold: first, to identify and explore views on appropriate and acceptable approaches to prevention across a range of stakeholders and, second, to determine how the intervention was implemented. In the former, local and national stakeholders involved in NTE governance, the sale of alcohol and the prevention of alcohol-related harm and disorder will be interviewed to identify obstacles, facilitators and acceptability of premises-level interventions. Results will inform the development of appropriate structures, resources and partnerships for any definitive trial. The latter component assessing intervention implementation and fidelity and any control arm contamination will allow the intervention to be refined for future iterations of the project and will facilitate the interpretation of outcome effects. An overview of the process evaluation plan is shown in Table 2.

**Table 2:** Description of the process evaluation

Group	Process Point & Method	Aims
Stakeholders in the NTE with respect to: • the governance of the NTE • the sale of alcohol • the prevention of alcohol related harm and disorder	Pre-intervention Semi-structured interview	• Views on intervention approaches and acceptability
Bar staff from premises in the study areas - not necessarily premises in the study	Pre-intervention Semi-structured interview	• Views on intervention approaches and acceptability
Premises Auditors/Street Surveyors	Post-intervention Focus Group	• Street survey protocols for future trials
Premises representative* (Intervention arm)	Post-intervention Semi-structured interview	• Views on intervention theory and acceptability • Contextual influences on implementation • Fidelity to action plans
Premises representative^1 ^(Control arm)	Post-intervention Semi-structured interview	• Assess contamination within double blind design

* Usually the Premises manager

## Results

### Recruitment

#### Phase 1: Letter of invitation

Recruitment through letters of invitation to all premises' Designated Premises Supervisors (DPS) in study areas produced low levels of interest: 1.5% of all premises' DPSs written to responded favourably.

#### Phase 2: Premises managers forum

Recruitment through presentation at fora where premises staff were present produced marginally higher rates of interest compared to written invitations yielding an estimated success rate of 6.2%.

#### Phase 3: Premises recruited with police

Recruitment through premises visits in the company of a police officer yielded a success rate of 100%.

#### Phase 4: Premises recruitment, no police

Recruiting through premises visits by a researcher who had been introduced to the premises by a police officer, but who did not attend with the researcher, yielded a success rate 100%.

### Sample, stratification and matching

Thirty-two premises were recruited for participation in the study (Town A: 6; Town B: 10; Town C: 8; Town D: 6; Town E: 2). Data used in calculating the risk index were generated acquired by accessing premises licences (publicly available through local council offices) and in consultation with premises representatives. Within towns, premises were matched by estimated risk score.

## Discussion

### Key issues in conducting the evaluation

#### Recruitment

in order to determine those resources required to recruit premises into a trial, recruitment methods of varying intensity were piloted. At the lowest intensity, premises were approached without reference to external agencies such as the police. This method did not yield a response rate sufficient for any future trial, suggesting that the problem premises cannot be relied on to voluntarily address alcohol-related harm. The only feasible recruitment method for a future trial would require the support of the police.

#### Stratification

Past premises-level interventions have not typically matched premises. Therefore premises features such as capacity and opening hours might affect results particularly when participant numbers are low. This study applied a simple risk index for matching premises (described above). However, as the sample size increases it is anticipated that matching will become less important [18].

#### Displacement and diffusion

Using licensed premises as the unit of allocation assumes that premises are independent. However, harm is realised in patrons and as such premises represent clusters of drinkers. As drinkers are free to move between premises, contamination between treatment arms is possible. For example, severe intoxication might become manifest at premises after excessive consumption even when no further consumption occurs at a second premises. Similarly, violence might erupt in a second premises but might partly reflect a fractious encounter at an earlier premises. Furthermore, a premises that successfully implements recommendations that curtail alcohol misuse and violence might have the effect of, rather than changing customers behaviour, encouraging those customers vulnerable to misuse and violence to relocate to premises more amenable to their proclivities. It is therefore critical that contamination and dispersal are appropriately monitored, and can be achieved through surveying. It is reasonable to assume that those who are most liable to misuse alcohol are those who have drunk heavily in the past [19,26,27], and that those who are most liable to be violent are those who have been violent in the past [28]. Thus, screening drinkers for their past experience of violence and taking FAST scores provide an appropriate means of assessing dispersal as these measures will describe patrons who visit a study premises. If an intervention shows an effect on, for example, alcohol misuse but this is attributable to a change in that premises clientele then changes in the aggregate FAST scores for that premises will highlight such dispersal effects. Further, asking patrons which premises they have visited and which premises they intend to visit also provides information on contamination.

#### Data quality

The reliability of police records of violence as a measure of all violence in and around a licensed premises is limited. A number of factors might adversely affect whether assaults are reported to the police. Some evidence suggests that the number of alcoholic drinks consumed prior to victimisation has a negative association with the likelihood of the reporting of violence by a victim [29]. However, this study was not specific to premises in the NTE and only considered the proclivity for victims to report the police when in the NTE police and premises staff might take responsibility for reporting an incident. Although bar staff, door staff and management might be aware that violent incidents in their premises that are known to the police can reflect badly on their reputation and continued trade, and could act as a disincentive for door staff to record and report incidents, NTEs typically have a visible police presence and are covered by CCTV. Thus, so long as any reporting biases are randomly allocated across treatment arms they are unlikely to influence the objectivity of a future trial.

## Conclusion

A definitive trial requires methods that provide sufficient premises to demonstrate a robust effect, the random allocation of treatment, and data at both the premises- and individual-level to assess outcomes. This exploratory trial provides sufficient detail to facilitate the development of such methods and together with a nested process evaluation informs our understanding of the acceptability of premises-levels interventions.

## Competing interests

The authors declare that they have no competing interests.

## Authors' contributions

SCM, SM and LM conceived of the study. SCM, SM, LM and JPS contributed to the original proposal from which this protocol was developed. SCM, SM, LM, EB, SNM and IRB were responsible for the conduct of the trial with SCM as principal investigator. IRB was responsible for the day to day management of the trial. EB and SNM took responsibility for and SM managed the process evaluation. SCM, IRB, SM, EB, LM and SM drafted the manuscript. All authors read and approved the final manuscript.

## Pre-publication history

The pre-publication history for this paper can be accessed here:

http://www.biomedcentral.com/1471-2458/10/607/prepub
